# Two-body decays of gluino at full one-loop level in the quark-flavour violating MSSM

**DOI:** 10.1140/epjc/s10052-017-4754-4

**Published:** 2017-03-25

**Authors:** Helmut Eberl, Elena Ginina, Keisho Hidaka

**Affiliations:** 1 Institut für Hochenergiephysik der Österreichischen Akademie der Wissenschaften, 1050 Vienna, Austria; 2 Department of Physics, Tokyo Gakugei University, Koganei, Tokyo, 184-8501 Japan

## Abstract

We study the two-body decays of the gluino at full one-loop level in the Minimal Supersymmetric Standard Model with quark-flavour violation (QFV) in the squark sector. The renormalisation is done in the $\overline{\mathrm{DR}}$ scheme. The gluon and photon radiations are included by adding the corresponding three-body decay widths. We discuss the dependence of the gluino decay widths on the QFV parameters. The main dependence stems from the $\tilde{c}_R$–$\tilde{t}_R$ mixing in the decays to up-type squarks, and from the $\tilde{s}_R$–$\tilde{b}_R$ mixing in the decays to down-type squarks due to the strong constraints from B-physics on the other quark-flavour-mixing parameters. The full one-loop corrections to the gluino decay widths are mostly negative and of the order of about −10%. The QFV part stays small in the total width but can vary up to −8% for the decay width into the lightest $\tilde{u}$ squark. For the corresponding branching ratio the effect is somehow washed out by at least a factor of two. The electroweak corrections can be as large as 35% of the SUSY QCD corrections.

## Introduction

After the discovery of the Higgs particle in 2012 [1, 2], a task with high priority of the LHC is the search for new physics, beyond the framework of the Standard Model (SM). One of the most favoured candidates to be discovered are the supersymmetric (SUSY) particles. Their decay chains have been, therefore, extensively studied during the last two decades. Especially relevant are the decays of strongly interacting SUSY particles, squarks and gluinos. At tree-level, the leading gluino decays are those into a quark and a squark. Only when these processes are kinematically forbidden, more-body and loop-induced gluino decays become important.

The decays of the gluino in the Minimal Supersymmetric Standard Model (MSSM) were previously studied with general quark-flavour violation (QFV) in the squark sector at tree level [3–5] or including one-loop corrections with no QFV in the squark sector [6, 7]. In this paper we study the two-body decays of the gluino into a scalar quark and a quark at full one-loop level with general quark-flavour mixing in the squark sector of the MSSM. Such a study has been performed in detail in [8]. The analytical results obtained therein, as well as the developed numerical package FVSFOLD, will be used in the current paper. Since the experiments on K-physics disfavour mixing between the first two squark generations [9], we only consider mixing between the second and the third generations of squarks. More concrete, we consider scenarios where the gluino only decays into the lightest up- and down-type squarks, $\tilde{u}_{1,2}$ and $\tilde{d}_{1,2}$, which can be mixtures of $\tilde{c}_{L,R}$ and $\tilde{t}_{L,R}$ and $\tilde{s}_{L,R}$ and $\tilde{b}_{L,R}$, respectively, and all the other decays into $\tilde{u}_{3,\ldots ,6}, \tilde{d}_{3,\ldots ,6}$ are kinematically forbidden. There exist constraints from B-physics on such mixing as well, which we take into account. The mass limits on SUSY particles as well as the theoretical constraints on the soft-SUSY-breaking trilinear coupling matrices from the vacuum stability conditions are also taken into account.

In Sect. 2 we give the formulae for the QFV mixing squark system. In Sect. 3 the tree-level partial two-body decay widths are derived and then the used $\overline{\mathrm{DR}}$ renormalisation scheme is explained. In order to cure the infrared (IR) divergences, we include the widths of the real gluon/photon radiation process, introducing a small regulator gluon/photon mass. In Sect. 4 we perform a detailed numerical analysis on the dependences of the two-body decay widths and branching ratios (BRs) on the quark-flavour-mixing parameters $\delta ^{uRR}_{23}$ and $\delta ^{dRR}_{23}$ and on the gluino mass. Appendix A contains the Lagrangian for the gluino–squark–quark interaction. In Appendix B all constraints we obey are summarised and Appendix C gives the detailed formulae for the hard radiation of a gluon or a photon.

## QFV parameters in the squark sector of the MSSM

We define the QFV parameters in the up-type squark sector of the MSSM as follows:

1 $$\begin{aligned} \delta ^{LL}_{\alpha \beta }\equiv & {} M^2_{Q \alpha \beta } / \sqrt{M^2_{Q \alpha \alpha } M^2_{Q \beta \beta }}, \end{aligned}$$

2 $$\begin{aligned} \delta ^{uRR}_{\alpha \beta }\equiv & {} M^2_{U \alpha \beta } / \sqrt{M^2_{U \alpha \alpha } M^2_{U \beta \beta }}, \end{aligned}$$

3 $$\begin{aligned} \delta ^{uRL}_{\alpha \beta }\equiv & {} (v_2/\sqrt{2} ) T_{U\alpha \beta } / \sqrt{M^2_{U \alpha \alpha } M^2_{Q \beta \beta }}, \end{aligned}$$

where $\alpha ,\beta =1,2,3 ~(\alpha \ne \beta )$ denote the quark flavours *u*, *c*, *t*, and $v_{2}=\sqrt{2} \left\langle H^0_{2} \right\rangle$. Analogously, for the down-type squark sector we have

4 $$\begin{aligned} \delta ^{dRR}_{\alpha \beta }\equiv & {} M^2_{D \alpha \beta } / \sqrt{M^2_{D \alpha \alpha } M^2_{D \beta \beta }}, \end{aligned}$$

5 $$\begin{aligned} \delta ^{dRL}_{\alpha \beta }\equiv & {} (v_1/\sqrt{2} ) T_{D\alpha \beta } / \sqrt{M^2_{D \alpha \alpha } M^2_{Q \beta \beta }}, \end{aligned}$$

where the subscripts $\alpha ,\beta =1,2,3 ~(\alpha \ne \beta )$ denote the quark flavours *d*, *s*, *b*, and $v_{1}=\sqrt{2} \left\langle H^0_{1} \right\rangle$. $M_{Q,U,D}$ are the hermitian soft-SUSY-breaking squark mass matrices and $T_{U,D}$ are the soft SUSY-breaking trilinear coupling matrices of the up- and down-type squarks. These parameters enter the left–left, right–right and left–right blocks of the $6\times 6$ squark mass matrix in the super-CKM basis [10],

6 $$\begin{aligned} \mathcal{M}^2_{\tilde{q}} = \left( \begin{array}{cc} \mathcal{M}^2_{\tilde{q},LL} &{} \mathcal{M}^2_{\tilde{q},LR} \\ \mathcal{M}^2_{\tilde{q},RL} &{} \mathcal{M}^2_{\tilde{q},RR} \end{array} \right) , \end{aligned}$$

with $\tilde{q}= \tilde{u}, \tilde{d}$. The different blocks in Eq. (6) are given by

7 $$\begin{aligned}&\mathcal{M}^2_{\tilde{u},LL} = V_\mathrm{CKM} M_Q^2 V_\mathrm{CKM}^{\dag } + D_{\tilde{u},LL}{} \mathbf{1} + \hat{m}^2_u, \nonumber \\&\mathcal{M}^2_{\tilde{u},RR} = M_U^2 + D_{\tilde{u},RR}{} \mathbf{1} + \hat{m}^2_u, \nonumber \\&\mathcal{M}^2_{\tilde{u},RL} = \mathcal{M}^{2\dag }_{\tilde{u},LR} = \frac{v_2}{\sqrt{2}} T_U - \mu ^* \hat{m}_u\cot \beta ,\nonumber \\&\mathcal{M}^2_{\tilde{d},LL} = M_Q^2 + D_{\tilde{d},LL}{} \mathbf{1} + \hat{m}^2_d, \nonumber \\&\mathcal{M}^2_{\tilde{d},RR} = M_D^2 + D_{\tilde{d},RR}{} \mathbf{1} + \hat{m}^2_d,\nonumber \\&\mathcal{M}^2_{\tilde{d},RL} = \mathcal{M}^{2\dag }_{\tilde{d},LR} = \frac{v_1}{\sqrt{2}} T_D - \mu ^* \hat{m}_d\tan \beta , \end{aligned}$$

where $\mu$ is the higgsino mass parameter, $\tan \beta$ is the ratio of the vacuum expectation values of the neutral Higgs fields $v_2/v_1$, and $\hat{m}_{u,d}$ are the diagonal mass matrices of the up- and down-type quarks. Furthermore, $D_{\tilde{q},LL} = \cos 2\beta m_Z^2 (T_3^q-e_q \sin ^2\theta _W)$ and $D_{\tilde{q},RR} = e_q \sin ^2\theta _W \cos 2\beta m_Z^2$, where $T_3^q$ and $e_q$ are the isospin and electric charge of the quarks (squarks), respectively, and $\theta _W$ is the Weinberg mixing angle. $V_\mathrm{CKM}$ is the Cabibbo–Kobayashi–Maskawa matrix, which we approximate with the unit matrix. The squark mass matrix is diagonalised by the $6\times 6$ unitary matrices $U^{\tilde{q}}$, such that

8 $$\begin{aligned}&U^{\tilde{q}} \mathcal{M}^2_{\tilde{q}} (U^{\tilde{q} })^{\dag } = \mathrm{diag}(m_{\tilde{q}_1}^2,\dots ,m_{\tilde{q}_6}^2), \end{aligned}$$

with $m_{\tilde{q}_1}< \dots < m_{\tilde{q}_6}$, and $\tilde{q}=\tilde{u}, \tilde{d}$. The physical mass eigenstates $\tilde{q}_i, i=1,\ldots ,6$ are given by $\tilde{q}_i = U^{\tilde{q}}_{i \alpha } \tilde{q}_{0\alpha }$.

In this paper we study $\tilde{c}_R$–$\tilde{t}_L$, $\tilde{c}_L$–$\tilde{t}_R$, $\tilde{c}_R$–$\tilde{t}_R$, and $\tilde{c}_L$–$\tilde{t}_L$ mixing, which is described by the QFV parameters $\delta ^{uRL}_{23}$, $\delta ^{uLR}_{23} \equiv ( \delta ^{uRL}_{32})^*$, $\delta ^{uRR}_{23}$, and $\delta ^{LL}_{23}$, respectively, as well as $\tilde{s}_R$–$\tilde{b}_L$, $\tilde{s}_L$–$\tilde{b}_R$, $\tilde{s}_R$–$\tilde{b}_R$, and $\tilde{s}_L$–$\tilde{b}_L$ mixing, which is described by the QFV parameters $\delta ^{dRL}_{23}$, $\delta ^{dLR}_{23} \equiv ( \delta ^{dRL}_{32})^*$, $\delta ^{dRR}_{23}$, and $\delta ^{LL}_{23}$, respectively. Note that $\delta ^{LL}_{23}$ describes the left–left mixing in both $\tilde{u}$ and $\tilde{d}$ sectors. The $\tilde{t}_R$–$\tilde{t}_L$ mixing is described by the quark-flavour conserving (QFC) parameter $\delta ^{uRL}_{33}$. All parameters mentioned are assumed to be real.

## Two-body decays of gluino at full one-loop level in the general MSSM

We study two-body decays of gluino into a squark and a quark, $\tilde{g}\rightarrow \tilde{q}^* q$. The tree-level partial decay widths $\Gamma ^0(\tilde{g}\rightarrow \tilde{q}^*_i q_g)$, with $i=1,\ldots ,6$, $q = u, d$, and the subscript *g* being the quark-generation index, are given by

9 $$\begin{aligned} \Gamma ^0(\tilde{g}\rightarrow \tilde{q}^*_i q_g) = {c\, \lambda ^{1/2}(m_{\tilde{g}}^2,m_{\tilde{q}_i}^2, m_{q_g}^2) \over 64\, \pi \, m_{\tilde{g}}^3}|\mathcal{M}_0|^2, \end{aligned}$$

where $c = 1/16$ is the average factor for the incoming $\tilde{g}$. The tree-level amplitude squared reads

10 $$\begin{aligned} |\mathcal{M}_0|^2= & {} (|g^i_L|^2+|g^i_R|^2)(m_{\tilde{g}}^2-m_{\tilde{q}_i}^2 +m_{q_g}^2) \nonumber \\&+\, 2 \,m_{\tilde{g}}m_{q_g}(g^{i*}_L g^i_R+g^i_L g_R^{i*}), \end{aligned}$$

with $\lambda (x^2,y^2, z^2) = x^2+y^2+z^2-2xy -2xz-2yz$, no summation over *i*, and the tree-level couplings $g^i_{L,R}$ are given by (see also Appendix A)

11 $$\begin{aligned} g^i_L=-\sqrt{2}~g_s T U^{\tilde{q}}_{i,g}, \quad g^i_R=\sqrt{2}~g_s T U^{\tilde{q}}_{i,g+3}, \end{aligned}$$

where *T* are the generators of the SU(3) colour group, and $U^{\tilde{q}}$, with $\tilde{q}=\tilde{u}, \tilde{d}$ are the up- and down-squark mixing matrices defined by Eq. (8). By inserting Eq. (11) into Eq. (10) and using tr$(T^a T^a) = N_c C_F = 4$ we can write Eq. (9) in the explicit form

12 $$\begin{aligned} \Gamma ^0(\tilde{g}\rightarrow \tilde{q}^*_i q_g)= & {} {\lambda ^{1/2}(m_{\tilde{g}}^2,m_{\tilde{q}_i}^2, m_{q_g}^2) \over 32\, m_{\tilde{g}}^3} \alpha _s \nonumber \\&\times \, \bigg (\left( |U^{\tilde{q}}_{i,g}|^2+|U^{\tilde{q}}_{i,g+3}|^2\right) (m_{\tilde{g}}^2-m_{\tilde{q}_i}^2 +m_{q_g}^2) \nonumber \\&- \, 4 m_{\tilde{g}}m_{q_g}\mathrm{Re}\left( U^{\tilde{q}*}_{i,g}\, U^{\tilde{q}}_{i,g+3} \right) \bigg ). \end{aligned}$$

In order to obtain an ultraviolet (UV) convergent result at one-loop level we employ the dimensional reduction ($\overline{\mathrm{DR}}$) regularisation scheme, which implies that all tree-level input parameters of the Lagrangian are defined at the scale $Q=M_3\approx m_{\tilde{g}}$. Since in this scheme the tree-level couplings $g^i_{L,R}$ are defined at the scale *Q*, they do not receive further finite shifts due to radiative corrections. The physical scale independent masses and fields are obtained from the $\overline{\mathrm{DR}}$ ones using on-shell renormalisation conditions.

We can write the renormalised one-loop partial decay widths as

13 $$\begin{aligned} \Gamma (\tilde{g}\rightarrow \tilde{q}^*_i q_g)= & {} \Gamma ^0( \tilde{g}\rightarrow \tilde{q}^*_i q_g)~+~\Delta \Gamma (\tilde{g}\rightarrow \tilde{q}^*_i q_g) ,\nonumber \\ \Delta \Gamma (\tilde{g}\rightarrow \tilde{q}^*_i q_g)= & {} {c\,\lambda ^{1/2}(m_{\tilde{g}}^2,m_{\tilde{q}_i}^2, m_{q_g}^2) \over 32\, \pi \, m_{\tilde{g}}^3} \mathrm{Re}(\mathcal{M}_0^\dagger \mathcal{M}_1),\nonumber \\ \mathrm{Re}(\mathcal{M}_0^\dagger \mathcal{M}_1)= & {} \mathrm{Re}\bigg ( (g^{i*}_L \Delta g_L + g^{i*}_R \Delta g_R)(m_{\tilde{g}}^2-m_{\tilde{q}_i}^2 +m_{q_g}^2) \nonumber \\&+ \, 2 m_{\tilde{g}}m_{q_g}(g^{i*}_L \Delta g_R + g^{i*}_R \Delta g_L)\bigg ), \end{aligned}$$

where $\mathcal{M}_1$ is the one-loop amplitude. The complete list of diagrams can be found in the appendix of [8]. The one-loop shifts to the coupling constants, $\Delta g_L$ and $\Delta g_R$, receive contributions from all vertex diagrams, the amplitudes arising from the wave-function renormalisation constants and the amplitudes arising from the coupling counter-terms,^1^

14 $$\begin{aligned} \Delta g_{L,R} = \delta g_{L,R}^v + \delta g_{L,R}^w +\delta g_{L,R}^c, \end{aligned}$$

where $\delta g_{L,R}^v$ is due to all vertex radiative corrections, and $\delta g_{L,R}^c$ is due to the coupling counter-terms. The wave-function induced corrections are given by

15 $$\begin{aligned} \delta g_{L}^{w, \mathrm{diag}}= & {} {1\over 2} \left( \delta Z^{\tilde{g}R*} + \delta Z_{ii}^{\tilde{q}*} +\delta Z_{gg}^{q L} \right) g_L^i,\nonumber \\ \delta g_{R}^{w, \mathrm{diag}}= & {} {1\over 2} \left( \delta Z^{\tilde{g}L*} + \delta Z_{ii}^{\tilde{q}*} +\delta Z_{gg}^{q R} \right) g_R^i, \nonumber \\ \delta g_{L}^{w, {\text{ off-diag }}}= & {} {1\over 2} \left( \delta Z_{ij}^{\tilde{q}*} g_L^j +\delta Z_{lg}^{q L} g_L^{i,l} \right) ,\nonumber \\ \delta g_{R}^{w, {\text{ off-diag }}}= & {} {1\over 2} \left( \delta Z_{ij}^{\tilde{q}*} g_R^j +\delta Z_{lg}^{q R} g_R^{i,l} \right) , \end{aligned}$$

with *i* and *j* fixed, $j \ne i$, $l \ne j$. Note that $g_{L,R}^{i,l}$ denote the $\tilde{g}\tilde{q}^{*}_i \bar{q}_l$ couplings. The explicit expressions for the renormalisation constants $\delta Z$ in (15) can be found in [8].

To cure the infrared (IR) divergences, in addition to (13), we include the widths of the real gluon/photon radiation processes, $\Gamma (\tilde{g}\rightarrow \tilde{q}_i q_g g /\gamma )$, assuming a small regulator gluon/photon mass $\lambda$. The explicit formulae for the hard radiation widths are given in Appendix C.

The full one-loop contribution to the total two-body decay width, see (13), is due to SUSY-QCD and electroweak corrections,

16 $$\begin{aligned} \Gamma (\tilde{g}\rightarrow \tilde{q}^* q)= & {} \Gamma ^0(\tilde{g}\rightarrow \tilde{q}^* q)+\Delta \Gamma ^\mathrm{SQCD}(\tilde{g}\rightarrow \tilde{q}^* q) \nonumber \\&+\, \Delta \Gamma ^\mathrm{EW}(\tilde{g}\rightarrow \tilde{q}^* q). \end{aligned}$$

$\Delta \Gamma ^\mathrm{SQCD}$ includes loops with gluon and gluino, and $\Delta \Gamma ^\mathrm{EW}$ includes loops with EW gauge bosons, photon, Higgs bosons and EWinos. In the numerical analyses performed in [8], as well as in [6], it was shown that in the considered scenarios the electroweak corrections are not negligible, but necessary for a correct one-loop evaluation. As you will see, in our numerical analysis we will come to a similar conclusion.

Hereafter we will use the notation $\Gamma (\tilde{g}\rightarrow \tilde{q}_i q_g) = \Gamma (\tilde{g}\rightarrow \tilde{q}^*_i q_g) + \Gamma (\tilde{g}\rightarrow \tilde{q}_i \bar{q}_g)$. In our case where CP is conserved this is equivalent with $2 \Gamma (\tilde{g}\rightarrow \tilde{q}^*_i q_g)$.

**Table 1:** QFV reference scenario: all parameters are calculated at $Q = M_3 = 3~\mathrm{TeV} \simeq m_{\tilde{g}}$, except for $m_{A^0}$ which is the pole mass of $A^0$, and $T_{U33} =2500$ GeV (corresponding to $\delta ^{uRL}_{33} = 0.06$). All other squark parameters are zero

$M_1$	$M_2$	$M_3$	$\mu$			
500 GeV	1000 GeV	3000 GeV	500 GeV			

$\tan \beta$	$m_{A^0}$					
15	3000 GeV					

	$\alpha = 1$	$\alpha = 2$	$\alpha = 3$			
$M_{Q \alpha \alpha }^2$	$3200^2~\mathrm{GeV}^2$	$3000^2~\mathrm{GeV}^2$	$2600^2~\mathrm{GeV}^2$			
$M_{U \alpha \alpha }^2$	$3200^2~\mathrm{GeV}^2$	$3000^2~\mathrm{GeV}^2$	$2600^2~\mathrm{GeV}^2$			
$M_{D \alpha \alpha }^2$	$3200^2~\mathrm{GeV}^2$	$3000^2~\mathrm{GeV}^2$	$2600^2~\mathrm{GeV}^2$			

$\delta ^{LL}_{23}$	$\delta ^{uRR}_{23}$	$\delta ^{uRL}_{23}$	$\delta ^{uLR}_{23}$	$\delta ^{dRR}_{23}$	$\delta ^{dRL}_{23}$	$\delta ^{dLR}_{23}$
0.01	0.7	0.04	0.07	0.7	0	0

## Numerical results

In order to demonstrate quantitatively our results on the gluino decay widths and branching ratios we first fix a reference scenario and then vary the QFV parameters within the allowed region. Our reference scenario fulfils all relevant theoretical and experimental constraints, which we discuss in more detail in Appendix B. The input parameters and the physical output parameters are shown in Tables 1 and 2, respectively. The flavour decomposition of the $\tilde{u}_{1,2}$ and $\tilde{d}_{1,2}$ squarks is shown in Table 3. For calculating the $h^0$ mass and the low-energy observables, especially those ones in the B-sector (see Table 4), we use the public code SPheno v3.3.3 [11, 12]. The gluino two-body widths and branching ratios at full one-loop level in the MSSM with QFV are calculated with the numerical code FVSFOLD, developed in [8]. For building FVSFOLD the packages FeynArts [13, 14] and FormCalc [15] were used. Furthermore, we use LoopTools [15] based on the FF package [16], and SSP [17]. In order to have simultanuosly a UV and IR finite result we first calculate the total result by using only ${\overline{\mathrm{DR}}}$ parameters for the one-loop partial width including the real hard radiation. Then we use on-shell masses, which are calculated within FVSFOLD, in the kinematic two-body prefactor $\lambda ^{1/2}/m_{\tilde{g}}^3$, see Eq. (12).

The scenario shown in Table 1 violates quark flavour explicitly in both up- and down-squark sectors. The values of the parameters $M_{1,2,3}$ are chosen to satisfy approximately the GUT relations ($M_1:M_2:M_3=1:2:6$). The Higgs boson $h^0$ is SM-like with $m_{h^0}=125~\mathrm{GeV}$ and all other Higgses are much heavier in mass and degenerate. The ratio of the vacuum expectation values of the neutral Higgs fields $v_2/v_1$ is taken relatively small, $\tan \mathop {=}\limits _{-}15$. The value of the $\mu$ parameter is also chosen small for naturalness reasons. The flavour decompositions of the $\tilde{u}_{1,2}$ and $\tilde{d}_{1,2}$ squarks are shown in Table 3. In this scenario the $\tilde{u}_1$ squark is a strong mixture of $\tilde{c}_R$ and $\tilde{t}_R$, with a tiny contribution from $\tilde{c}_L$, and the $\tilde{u}_2$ squark is mainly $\tilde{t}_L$, with a tiny contribution from $\tilde{c}_R$. The $\tilde{d}_1$ is a mixture of $\tilde{s}_R$ and $\tilde{b}_R$, and $\tilde{d}_2$ is a pure $\tilde{b}_L$.

**Table 2:** Physical masses of the particles in GeV for the scenario of Table 1

$m_{\tilde{\chi }^0_{1}}$	$m_{\tilde{\chi }^0_{2}}$	$m_{\tilde{\chi }^0_{3}}$	$m_{\tilde{\chi }^0_{4}}$	$m_{\tilde{\chi }^+_{1}}$	$m_{\tilde{\chi }^+_{2}}$	
460	500	526	1049	493	1049	

$m_{h^0}$	$m_{H^0}$	$m_{A^0}$	$m_{H^+}$			
125	3000	3000	3001			

$m_{\tilde{g}}$	$m_{\tilde{u}_{1}}$	$m_{\tilde{u}_{2}}$	$m_{\tilde{u}_{3}}$	$m_{\tilde{u}_{4}}$	$m_{\tilde{u}_{5}}$	$m_{\tilde{u}_{6}}$
3154	1602	2686	3087	3295	3300	3692

$m_{\tilde{d}_{1}}$	$m_{\tilde{d}_{2}}$	$m_{\tilde{d}_{3}}$	$m_{\tilde{d}_{4}}$	$m_{\tilde{d}_{5}}$	$m_{\tilde{d}_{6}}$	
1662	2689	3087	3295	3301	3747	

**Table 3:** Flavour decomposition of $\tilde{u}_{1,2}$ and $\tilde{d}_{1,2}$ for the scenario of Table 1. Shown are the squared coefficients

	$\tilde{u}_L$	$\tilde{c}_L$	$\tilde{t}_L$	$\tilde{u}_R$	$\tilde{c}_R$	$\tilde{t}_R$
$\tilde{u}_1$	0	0.004	0	0	0.38	0.61
$\tilde{u}_2$	0	0.001	0.99	0	0.006	0

	$\tilde{d}_L$	$\tilde{s}_L$	$\tilde{b}_L$	$\tilde{d}_R$	$\tilde{s}_R$	$\tilde{b}_R$
$\tilde{d}_1$	0	0	0	0	0.4	0.6
$\tilde{d}_2$	0	0	1	0	0	0

At our reference parameter point the gluino decays into $\tilde{u}_{1,2}\,c$, $\tilde{u}_{1,2} \,t$, $\tilde{d}_{1,2} \,s$ and $\tilde{u}_{1,2} \,b$ are kinematically allowed, with branching ratios B$(\tilde{g}\rightarrow \tilde{u}_1\,c)\approx 17\%$, B$(\tilde{g}\rightarrow \tilde{d}_1\,s)\approx 18\%$, B$(\tilde{g}\rightarrow \tilde{u}_1\,t) = \mathrm{B}(\tilde{g}\rightarrow \tilde{d}_1\,b) \approx 27\%$, B$(\tilde{g}\rightarrow \tilde{u}_2\,t)\approx 5\%$ and the others being very small. The total two-body width including the full one-loop contribution, $\Gamma (\tilde{g}\rightarrow \tilde{q}q) = 70\,\mathrm{GeV}$, where the tree-level width $\Gamma ^0(\tilde{g}\rightarrow \tilde{q}q) = 75\,\mathrm{GeV}$,^2^ and the SUSY-QCD and the electroweak corrections are negative, $\Delta \Gamma ^\mathrm{SQCD}(\tilde{g}\rightarrow \tilde{q}q) = -4.6\,\mathrm{GeV}$ and $\Delta \Gamma ^\mathrm{EW}(\tilde{g}\rightarrow \tilde{q}q) = -0.5\,\mathrm{GeV}$, giving about −6.4 and −0.7% of the total two-body gluino width $\Gamma (\tilde{g}\rightarrow \tilde{q}q)$, respectively. Note that the SQCD contribution includes gluon and gluino, and the EW contribution includes also the photon. In the same scenario with no quark-flavour violation, i.e. when all QFV ($\delta$) parameters listed in Table 1 are set to zero, we have $\Gamma (\tilde{g}\rightarrow \tilde{q}q) = 14\,\mathrm{GeV}$.

**Fig. 1:**
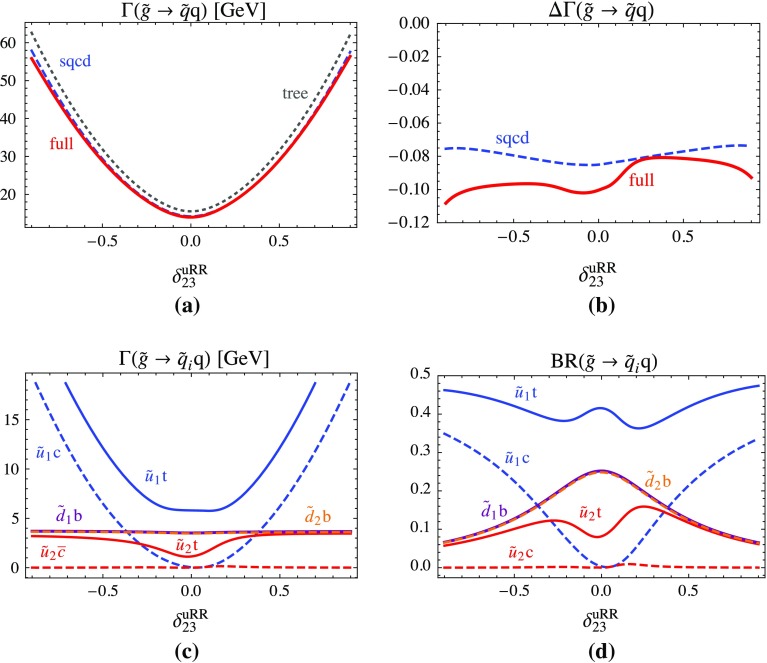
**a** Total two-body decay width $\Gamma (\tilde{g}\rightarrow \tilde{q}q)$ at tree level, SQCD one-loop and full one-loop corrected as functions of the QFV parameter $\delta ^{uRR}_{23}$; **b** $\Delta \Gamma (\tilde{g}\rightarrow \tilde{q}q)$ being the SQCD one-loop and the full one-loop corrections to $\Gamma (\tilde{g}\rightarrow \tilde{q}q)$ relative to the tree-level width; **c** partial decay widths and **d** branching ratios of the kinematically allowed individual two-body channels at full one-loop level as functions of $\delta ^{uRR}_{23}$. All the other parameters are fixed as in Table 1, except $\delta ^{uRL}_{23}= \delta ^{uLR}_{23}= 0.03$

The QFV left–right mixing, described by the parameters $\delta ^{uLR}_{23}, \delta ^{uRL}_{23}, \delta ^{dLR}_{23}, \delta ^{dRL}_{23}$, is constrained from the vacuum stability conditions (see Sect. B) and is required to be rather small. On another hand, a sizeable value of $\delta ^{LL}_{23}$ is not possible because it violates B-physics constraints such as the B($B_s \rightarrow \mu ^+ \mu ^-$) constraint. However, large right–right mixing in both $\tilde{u}$ and $\tilde{d}$ sectors is allowed (see also [18]) and therefore, in the following, we show only plots with dependences on the $\delta ^{uRR}_{23}$ and $\delta ^{dRR}_{23}$ parameters. In Fig. 1 we show dependences on the QFV parameter $\delta ^{uRR}_{23}$. In Fig. 1a the tree-level, the SQCD and total full one-loop widths and in Fig. 1b the relative contributions of the one-loop SQCD and the full one-loop part in terms of the tree-level result are shown. The partial decay widths as well as the branching ratios of the kinematically allowed two-body channels at full one-loop level are shown in Fig. 1c, d, respectively. In Fig. 1a it is seen that $\Gamma (\tilde{g}\rightarrow \tilde{q}q)$ is quite sensitive to the parameter $\delta ^{uRR}_{23}$. The dependence of the tree-level width and the full one-loop corrected width is similar and their difference becomes a little more important for large absolute values of $\delta ^{uRR}_{23}$. This means that the QFV parameter dependence is mainly due to the kinematic factor, see Sect. 3. The SQCD correction shown in Fig. 1b is only weakly dependent on $\delta ^{uRR}_{23}$ and is about −8%. The EW correction can become −3% for large and negative values of $\delta ^{uRR}_{23}$. In Fig. 1c the partial widths of the $\tilde{d}_{1,2} b$ modes coincide because $m_{\tilde{d}_1}\approx m_{\tilde{d}_2}$. The same holds for the branching ratios in Fig. 1d. For $\delta ^{uRR}_{23}\approx 0$ the width of $\tilde{g} \rightarrow \tilde{u}_1 c$ becomes tiny because then $\tilde{u}_1$ is mainly $\tilde{t}_R$ as all the other QFV $\delta$’s are relatively small.

**Fig. 2:**
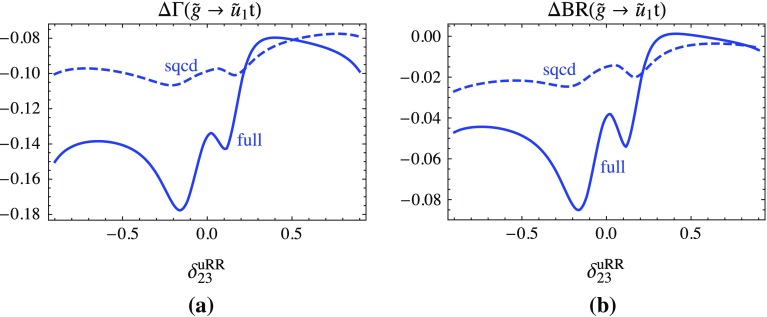
$\Delta \Gamma$ and $\Delta$BR denote the SQCD one-loop and the full one-loop corrections relative to the tree-level result for the decay $\tilde{g}\rightarrow \tilde{u}_1 t$ as a function of $\delta ^{uRR}_{23}$; **a**, **b** is for the partial width and the branching ratio, respectively. The other parameters are fixed as in Fig. 1

Figure 2 shows the relative contribution of the one-loop SQCD and the full one-loop part in terms of the tree-level result for the partial decay width (Fig. 2a) and the branching ratio (Fig. 2b) of the decay $\tilde{g} \rightarrow \tilde{u}_1 t$ as a function of $\delta ^{uRR}_{23}$. We see in Fig. 2a that the SQCD corrections vary in the range of −8 to −10%. The EW correction is much stronger dependent on $\delta ^{uRR}_{23}$ varying between 1% down to −8%. The effects are similar in the branching ratio (b), but weaker. Out of the squark masses only $m_{\tilde{u}_1}$ is strongly dependent on $\delta ^{uRR}_{23}$. In the whole range of $\delta ^{uRR}_{23}$ no additional channel opens but those visible in Fig. 1c, d. Therefore, the wiggles stem from the complex structures of the QFV one-loop contributions.

**Fig. 3:**
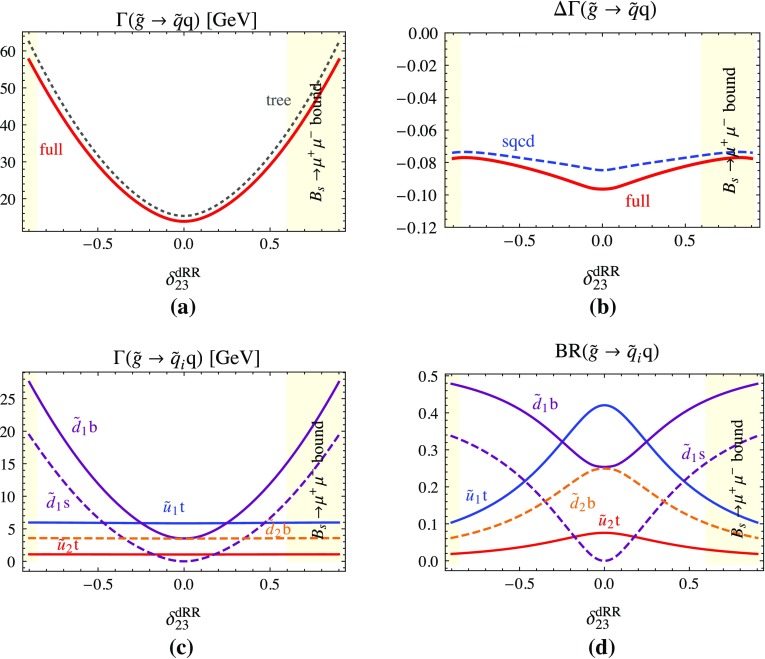
**a** Total two-body decay width $\Gamma (\tilde{g}\rightarrow \tilde{q}q)$ at tree level and full one-loop corrected (which coincides with the SQCD one-loop corrected one) as functions of the QFV parameter $\delta ^{dRR}_{23}$; **b** $\Delta \Gamma (\tilde{g}\rightarrow \tilde{q}q)$ being the SQCD one-loop and the full one-loop corrections to $\Gamma (\tilde{g}\rightarrow \tilde{q}q)$ relative to the tree-level width; **c** partial decay widths and **d** branching ratios of the kinematically allowed individual two-body channels at full one-loop level as functions of $\delta ^{dRR}_{23}$. All the other parameters are fixed as in Table 1, except $\delta ^{uRL}_{23}= \delta ^{uLR}_{23}=\delta ^{uRR}_{23}= 0$

In Fig. 3 we show dependences on the QFV parameter $\delta ^{dRR}_{23}$. In Fig. 3a the tree-level, the SQCD and total full one-loop widths and in Fig. 3b the relative contribution of the one-loop SQCD and the full one-loop part in terms of the tree-level result are shown. The partial decay widths as well as the branching ratios of the kinematically allowed two-body channels are shown in Fig. 3c, d, respectively. A comparison of Fig. 3 with Fig. 1 demonstrates the equal importance of QFV mixing in both $\tilde{u}$ and $\tilde{d}$ sector. But in the $\tilde{d}$ sector all plots are more symmetric around $\delta ^{dRR}_{23}= 0$ compared to these in $\tilde{u}$ sector around $\delta ^{uRR}_{23}= 0$. This stems from the fact that in the $\tilde{u}$ mass matrix $T_{U33} = 2500$ GeV but in the $\tilde{d}$ mass matrix $T_{D33} = 0$ GeV is taken and $m_b\, \mu \tan \beta$ is relatively small, see Eq. (7). The SQCD corrected width in Fig. 3a seem to coincide with the full one-loop corrected width, which we see in detail in Fig. 3b. There the SQCD correction is about −7.5% and varies only within 1% around this value. The EW part varies between −0.5 to −1.5%. In the Fig. 3c, d we see that for large absolute values of the $\tilde{d}$ right–right mixing parameter $\delta ^{dRR}_{23}$ the $\tilde{d}_1$ decay modes become much more important than the $\tilde{u}$ ones since the $\tilde{d}_1$ mass becomes smaller due to the mixing effect. As $\tilde{d}_{1,2}$ are mainly bottom squarks, the EW corrections to the $\tilde{d}_{1,2} b$ modes are small, mainly controlled by the rather small bottom-quark Yukawa coupling $Y_b(Q = 3~\mathrm{TeV})$ for $\tan \mathop {=}\limits _{-} 15$. On the other hand, as $\tilde{u}_{1,2}$ are mainly top squarks, the EW corrections to the $\tilde{u}_{1,2} t$ modes are significant, mainly controlled by the large top-quark Yukawa coupling $Y_t$. This explains the smallness of the EW corrections in Fig. 3a, b, especially for large $|\delta ^{dRR}_{23}|$.

**Fig. 4:**
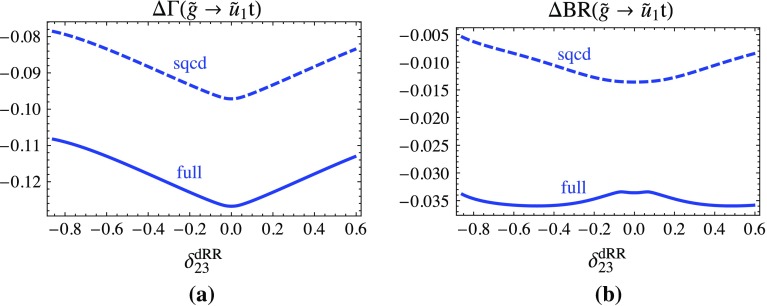
$\Delta \Gamma$ and $\Delta$BR denote the SQCD and the full one-loop contribution in terms of the tree-level result for the decay $\tilde{g}\rightarrow \tilde{u}_1 t$ as a function of $\delta ^{dRR}_{23}$, **a** to the partial width, **b** to the branching ratio, respectively. The parameters are fixed as in Fig. 3

Figure 4 shows the relative contribution of the one-loop SQCD and the full one-loop part in terms of the tree-level result for the partial decay width Fig. 4a and the branching ratio Fig. 4b of the decay $\tilde{g} \rightarrow \tilde{u}_1 t$ as a function of $\delta ^{dRR}_{23}$ in the phenomenologically allowed region. The interesting point is that the dependence of this channel on $\delta ^{dRR}_{23}$ comes mainly from the gluino wave-function correction term with $\tilde{d}$ in the loop. The SQCD correction varies between −8 and −9.5% and the EW correction is about constant and is $\sim -3$% for the width (Fig. 4a). For the branching ratio (Fig. 4b), the effect is much smaller for the SQCD correction, between −0.5 and −1.5%. The EW part is maximal −3%.

**Fig. 5:**
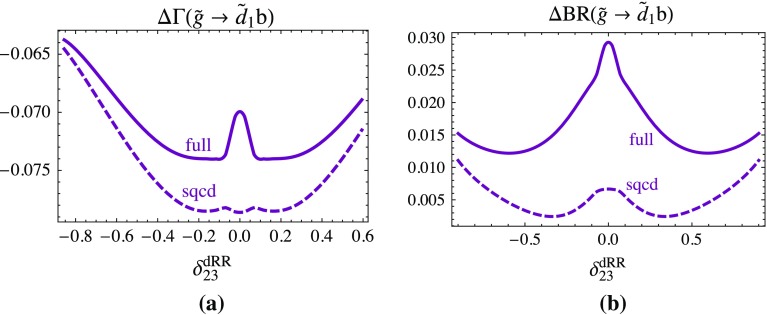
$\Delta \Gamma$ and $\Delta$BR denote the SQCD and the full one-loop contribution in terms of the tree-level result for the decay $\tilde{g}\rightarrow \tilde{d}_1 b$ as a function of $\delta ^{dRR}_{23}$, **a** to the partial width, **b** to the branching ratio, respectively. The parameters are fixed as in Fig. 3

Figure 5 shows the relative contribution of the one-loop SQCD and the full one-loop part in terms of the tree-level result for the partial decay width (Fig. 5a) and the branching ratio (Fig. 5b) of the decay $\tilde{g} \rightarrow \tilde{d}_1 b$ as a function of $\delta ^{dRR}_{23}$ in the phenomenologically allowed region. The SQCD correction varies between −6.5 and −8% and the EW correction can become $\sim$1% for the width (Fig. 5a). For the branching ratio (Fig. 5b), the effects are again smaller, the SQCD correction is less than 1% and the EW part maximal 3%. As in Fig. 2 the wiggles stem from the complex structures of the QFV one-loop contributions.

**Fig. 6:**
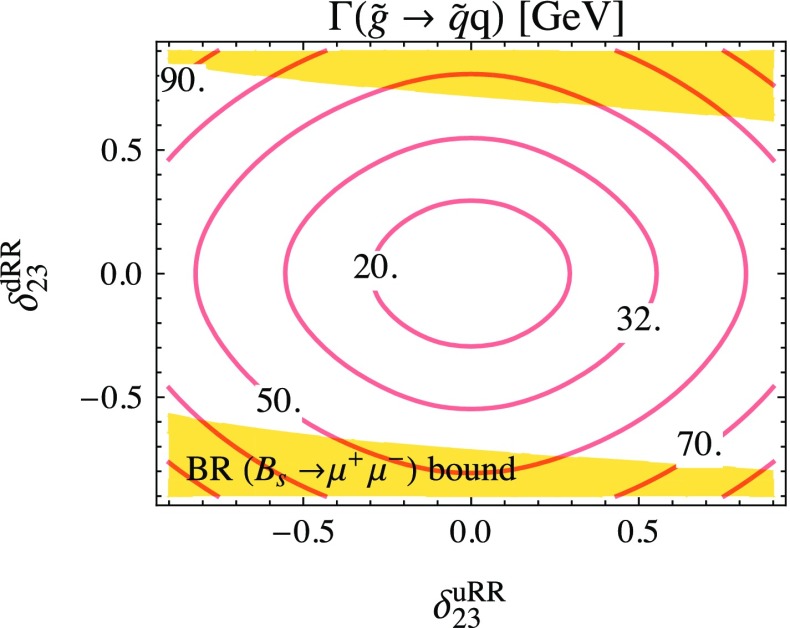
Total two-body decay width $\Gamma (\tilde{g}\rightarrow \tilde{q}q)$ at full one-loop level as a function of the QFV parameters $\delta ^{dRR}_{23}$ and $\delta ^{uRR}_{23}$. All the other parameters are given in Table 1, except $\delta ^{uRL}_{23}= \delta ^{uLR}_{23}= 0.01$

In Fig. 6 a simultaneous dependence on the right–right mixing parameters of both $\tilde{u}$ and $\tilde{d}$ sectors is shown. It is clearly seen that the total two-body decay width $\Gamma (\tilde{g}\rightarrow \tilde{q}q)$ can vary up to 70 GeV in the allowed parameter region due to QFV.

**Fig. 7:**
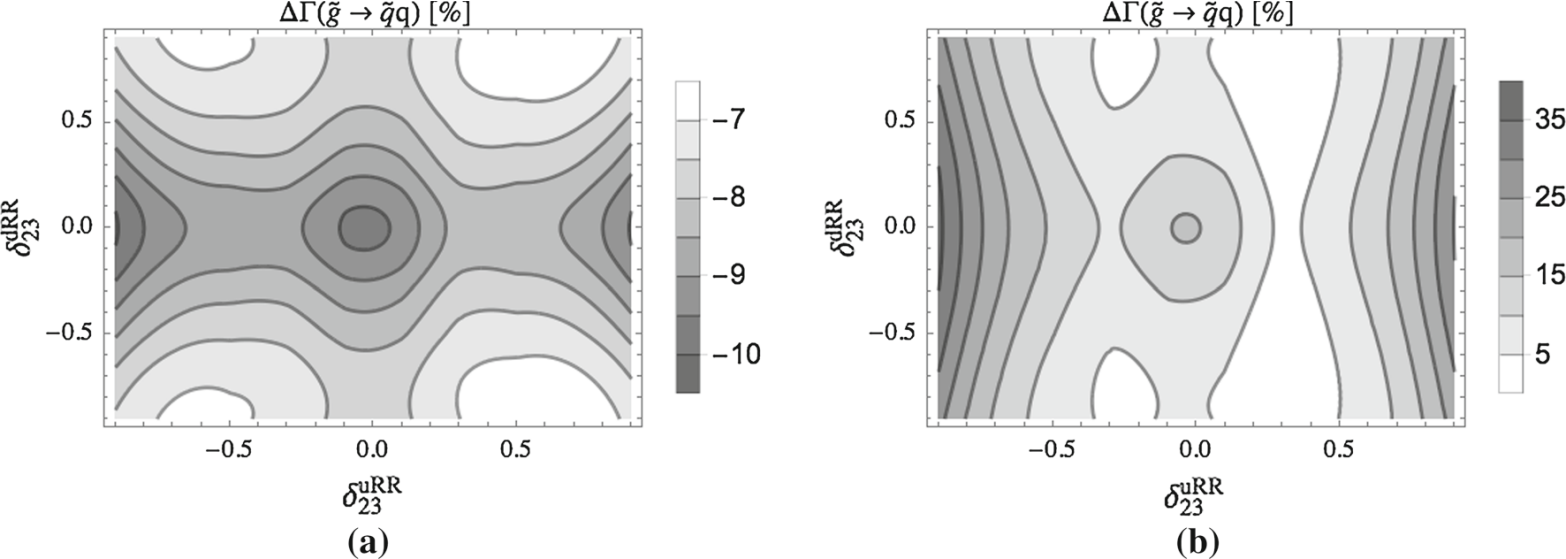
$\Delta \Gamma$ denotes in **a** the full one-loop contribution in terms of the total tree-level width, in **b** the EW contribution relative to the SQCD contribution. Both *plots* are given as a function of the QFV parameters $\delta ^{uRR}_{23}$ and $\delta ^{dRR}_{23}$. All the other parameters are given in Table 1, except $\delta ^{uRL}_{23}= \delta ^{uLR}_{23}= 0.01$

In Fig. 7a the full one-loop part in terms of the tree-level result and in Fig. 7b the EW contribution relative to the SQCD contribution are shown for the total two-body gluino decay width as a function of $\delta ^{uRR}_{23}$ and $\delta ^{dRR}_{23}$. We see in Fig. 7a a constant QFC one-loop contribution of $\sim -10$ and $\sim 3$% variation due to QFV. The EW part can become up to $\sim$35% of the SQCD one (Fig. 7b) for large $|\delta ^{uRR}_{23}|$ where the $\tilde{u}_1 t$ mode becomes important, since the $\tilde{u}_1$ mass becomes smaller due to the $\tilde{u}$-sector right–right mixing effect. Furthermore, as $\tilde{u}_1$ is mainly a top squark, the EW corrections to the $\tilde{u}_1 t$ mode are significant, mainly controlled by the large top-quark Yukawa coupling $Y_t$.

**Fig. 8:**
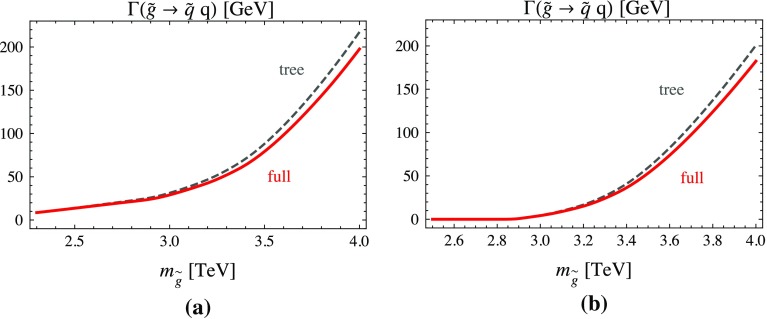
Dependence of the total two-body decay width $\Gamma (\tilde{g}\rightarrow \tilde{q}q)$ at tree level (*dashed*) and full one-loop level (*solid*) on the gluino mass. **a** QFV scenario with the parameters as given in Table 1; **b** QFC scenario with the parameters as given in Table 1, but with all QFV ($\delta$) parameters set to zero

Figure 8 shows the dependence of the total two-body decay width $\Gamma (\tilde{g}\rightarrow \tilde{q}q)$ on the gluino mass in our reference scenario (Fig. 8a) and in a quark-flavour conserving scenario, setting all QFV ($\delta$) parameters of Table 1 to zero (Fig. 8b). It is seen that in the QFV scenario (Fig. 8a) $\Gamma (\tilde{g}\rightarrow \tilde{q}q)$ is somewhat enhanced. Because of the large $|\delta ^{uRR}_{23}|$ ($|\delta ^{dRR}_{23}|$) the mass difference between $\tilde{u}_{1}$ and $\tilde{u}_{2}$ ($\tilde{d}_{1}$ and $\tilde{d}_{2}$) is bigger. Consequently, $\tilde{u}_1$ and $\tilde{d}_1$ are lighter and decays into these particles are already possible for smaller gluino masses.

We have compared our numerical results in the flavour conserving limit with the results obtained in [6]. For their reference scenario with $M_3 = 2000$ GeV assuming their input parameters to be $\overline{\mathrm{DR}}$ ones, we get a total width of 379 GeV. We agree with them within 2%. For the relative size of the full one-loop correction we get −2% compared to their result of −2.5%.

## Conclusions

We have studied all two-body decays of the gluino at full one-loop level in the Minimal Supersymmetric Standard Model with quark-flavour violation in the squark sector. We have discussed a scenario where only the decays to $\tilde{u}_{1,2}$ and $\tilde{d}_{1,2}$ are kinematically open and $\tilde{u}_1$ is a mixture of $\tilde{c}_R$ and $\tilde{t}_R$ controlled by $\delta _{23}^{uRR}$, and $\tilde{d}_1$ is a mixture of $\tilde{s}_R$ and $\tilde{b}_R$ controlled by $\delta _{23}^{dRR}$. All other QFV parameters are small in order to fulfil the constraints from B-physics. The LHC constraints for the masses of the SUSY particles are also satisfied, especially that one for $m_{h^0}$ and the vacuum stability conditions are fulfilled.

The full one-loop corrections to the gluino decay widths are mostly negative. For the total decay width they are in the range of −10% with a weak dependence on QFV parameters for both SQCD (including gluon loops) and electroweak (including also photon loops) corrections. For the decay width into $\tilde{u}_1$ we can have a total correction up to −18%, with the EW part up to −8%, strongly depending on the QFV parameters. For the corresponding branching ratio the effect is somehow washed out. For the decay into $\tilde{d}_1$ we have maximal corrections of −8%. In general, it turns out that the EW corrections can be in the range of up to 35% of the SQCD corrections due to the large top-quark Yukawa coupling. The full one-loop corrections to the total width are of the order of about −10% in the gluino mass range of 2.3–4.0 TeV.

## Figures and Tables

**Fig. 9:**
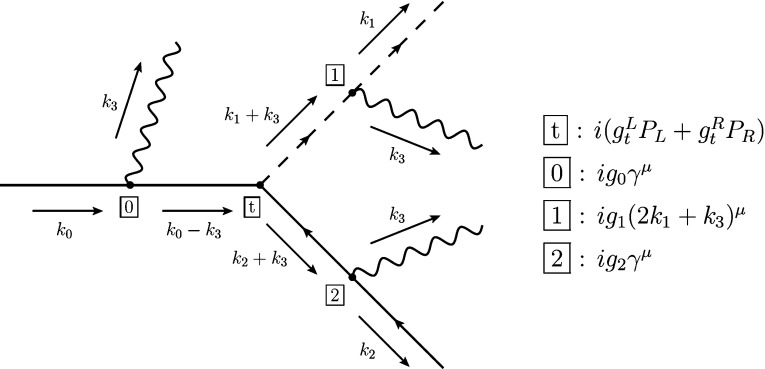
The combination of three Feynman graphs for the $1 \rightarrow 3$ bremsstrahlung process emitting a photon or a gluon from a fermion to scalar–fermion structure

## Interaction Lagrangian

The interaction of gluino, squark and quark is given by

**Table 4:** Constraints on the MSSM parameters from the B-physics experiments relevant mainly for the mixing between the second and the third generations of squarks and from the data on the $h^0$ mass. The fourth column shows constraints at 95% CL obtained by combining the experimental error quadratically with the theoretical uncertainty, except for $m_{h^0}$

Observable	Exp. data	Theor. uncertainty	Constr. (95% CL)
$\Delta M_{B_s}$ [ps$^{-1}$]	$17.757 \pm 0.021$ (68% CL) [19]	$\pm 3.3$ (95% CL) [20, 21]	$17.757 \pm 3.30$
$10^4\times$B($b \rightarrow s \gamma )$	$3.41 \pm 0.155$ (68% CL) [22]	$\pm 0.23$ (68% CL) [23]	$3.41\pm 0.54$
$10^6\times$B($b \rightarrow s~l^+ l^-$)	$1.60^{+0.48}_{-0.45}$ (68% CL) [24]	$\pm 0.11$ (68% CL) [25]	$1.60^{+0.97}_{-0.91}$
$$(l=e~\mathrm{or}~\mu )$$			
$10^9\times$B($B_s\rightarrow \mu ^+\mu ^-$)	$2.8^{+0.7}_{-0.6}$ (68% CL) [26]	$\pm 0.23$ (68% CL) [27]	$2.80^{+1.44}_{-1.26}$
$10^4\times$B($B^+ \rightarrow \tau ^+ \nu$)	$1.14 \pm 0.27$ (68% CL) [22, 28]	$\pm 0.29$ (68% CL) [29]	$1.14 \pm 0.78$
$$ m_{h^0}$$ [GeV]	$$125.09 \pm 0.24~(68\%~ \mathrm {CL})$$ [30]	$$\pm 3$$ [31]	$$125.09 \pm 3.48$$

17 $$\begin{aligned} \mathcal{L}_{\tilde{g}\tilde{q}_i q_g}= & {} -\sqrt{2} g_s T_{rs}^{\alpha }\bigg [\bar{\tilde{g}}^{\alpha }(U^{\tilde{q}}_{i,g}e^{-i\frac{\phi _3}{2}} P_L-U^{\tilde{q}}_{i,g+3}e^{i\frac{\phi _3}{2}}P_R) q_g^s \tilde{q}_i^{*, r} \nonumber \\&+ \, \bar{q}_g^r (U^{\tilde{q}*}_{i,g} e^{i\frac{\phi _3}{2}}P_R-U^{\tilde{q}*}_{i,g+3}e^{-i\frac{\phi _3}{2}} P_L) \tilde{g}^{\alpha } \tilde{q}_i^s\bigg ], \end{aligned}$$

where $T^\alpha$ are the SU(3) colour group generators, g is the generation index ($g=u,c,t$ for up-type quarks and $g=d,s,b$ for down-type quarks), and summation over $r,s=1,2,3$ and over $\alpha =1,\ldots ,8$ is understood. In our case the parameter $M_3=m_{\tilde{g}}e^{i \phi _3}$ is taken to be real, *i.e.* $\phi _3=0$.

## Theoretical and experimental constraints

Here we summarise the experimental and theoretical constraints taken into account in the present paper. The constraints on the MSSM parameters from the B-physics experiments and from the Higgs boson measurement at LHC are shown in Table 4. The constraints from the decays $B\rightarrow D^{(*)}\,\tau \,\nu$ are unclear due to large theoretical uncertainties [32]. Therefore, we do not take these constraints into account in our paper. In [33, 34] it is shown that the QFV decay $t \rightarrow c\,h^0$ in the current LHC runs cannot give any significant constraint on the $\tilde{c}$–$\tilde{t}$ mixing.

For the mass of the Higgs boson $h^0$, taking the combination of the ATLAS and CMS measurements $m_{h^0} = 125.09 \pm 0.24~\mathrm{GeV}$ [30] and adding the theoretical uncertainty of $\sim \pm 3~\mathrm{GeV}$  [31] linearly to the experimental uncertainty at 2 $\sigma$, we take $m_{h^0} = 125.09 \pm 3.48 ~\mathrm{GeV}$.

In addition to these constraints we also require our scenarios to be consistent with the following experimental constraints:

(i) The LHC limits on the squark and gluino masses (at 95% CL)  [35]:

In the context of simplified models, gluino masses $m_{\tilde{g}}\lesssim 1.9~\mathrm{TeV}$ are excluded at 95% CL. The mass limit varies in the range 1400–1900 GeV depending on assumptions. First and second generation squark masses are excluded below 1400 GeV. Bottom squark masses are excluded below 1000 GeV. A typical top-squark mass limit is $\sim$900 GeV.

(ii) The LHC limits on $m_{\tilde{\chi }^\pm _1}$ and $m_{\tilde{\chi }^0_2}$ from negative searches for charginos and neutralinos mainly in leptonic final states  [35].
(iii) The constraint on ($m_{A^0, H^+} , \tan \beta$) from the MSSM Higgs boson searches at LHC  [36, 37].
(iv) The experimental limit on SUSY contributions on the electroweak $\rho$ parameter  [38]: $\Delta \rho ~ (\mathrm SUSY) < 0.0012.$

Furthermore, we impose the following theoretical constraints from the vacuum stability conditions for the trilinear coupling matrices [39]:

18 $$\begin{aligned} |T_{U\alpha \alpha }|^2< & {} 3~Y^2_{U\alpha }~(M^2_{Q \alpha \alpha }+M^2_{U\alpha \alpha }+m^2_2)~, \end{aligned}$$

19 $$\begin{aligned} |T_{D\alpha \alpha }|^2< & {} 3~Y^2_{D\alpha }~(M^2_{Q\alpha \alpha }+M^2_{D\alpha \alpha }+m^2_1)~, \end{aligned}$$

20 $$\begin{aligned} |T_{U\alpha \beta }|^2< & {} Y^2_{U\gamma }~(M^2_{Q \beta \beta }+M^2_{U\alpha \alpha }+m^2_2)~, \end{aligned}$$

21 $$\begin{aligned} |T_{D\alpha \beta }|^2< & {} Y^2_{D\gamma }~(M^2_{Q \beta \beta }+M^2_{D\alpha \alpha }+m^2_1)~, \end{aligned}$$

where $\alpha ,\beta =1,2,3,~\alpha \ne \beta ;~\gamma =\mathrm{Max}(\alpha ,\beta )$ and $m^2_1=(m^2_{H^+}+m^2_Z\sin ^2\theta _W)\sin ^2\beta -\frac{1}{2}m_Z^2$, $m^2_2=(m^2_{H^+}+$ $m^2_Z\sin ^2\theta _W)$ $\cos ^2\beta -\frac{1}{2}m_Z^2$. The Yukawa couplings of the up-type and down-type quarks are $Y_{U\alpha }=\sqrt{2}m_{u_\alpha }/v_2=\frac{g}{\sqrt{2}}\frac{m_{u_\alpha }}{m_W \sin \beta }$ $(u_\alpha =u,c,t)$ and $Y_{D\alpha }=\sqrt{2}m_{d_\alpha }/v_1=\frac{g}{\sqrt{2}}\frac{m_{d_\alpha }}{m_W \cos \beta }$ $(d_\alpha =d,s,b)$, with $m_{u_\alpha }$ and $m_{d_\alpha }$ being the running quark masses at the weak scale and *g* being the SU(2) gauge coupling. All soft-SUSY-breaking parameters are given at $\mathrm Q=3$ TeV. As SM parameters we take $m_Z=91.2~\mathrm{GeV}$ and the on-shell top-quark mass $m_t=173.3~\mathrm{GeV}$ [40].

## Hard photon/gluon radiation

We start with the general formula of a $1 \rightarrow 3$ process with the hard radiation of a photon or a gluon,

22 $$\begin{aligned} \Gamma ^\mathrm{hard}= & {} {1 \over 2^6 m_0 \pi ^3} \int {\mathrm{d}^3k_1 \over 2 E_1} \nonumber \\&\times {\mathrm{d}^3k_2 \over 2 E_2} {\mathrm{d}^3k_3 \over 2 E_3} \delta ^4(k_0 - k_1 - k_2 - k_3) \overline{|\mathcal{M}^\mathrm{hard} |^2} . \end{aligned}$$

The bar means we take the average of incoming spins and colours and sum over the outgoing spins and colours. Based on the diagram (Fig. 9) and using the definition of the bremsstrahlung’s integrals from [41] we can write Eq. (22) as

23 $$\begin{aligned} \Gamma ^\mathrm{hard} = {col \over 2^6 m_0 \pi ^3} X_\mathrm{FSF}, \end{aligned}$$

where *col* denotes the colour average of the incoming particle and the fermion to scalar–fermion structure factor

24 $$\begin{aligned} X_\mathrm{FSF}= & {} g_0^2 \bigg [ \left( -2 \alpha m_0^2 -2 \beta m_2 m_0 \right) I_0 - \alpha I_0^2 \nonumber \\&+\, \left( -2 \alpha \left( m_0^2 -m_1^2 +m_2^2\right) m_0^2 - 4 \beta m_2 m_0^3 \right) I_{00}\bigg ] \nonumber \\&+ \, g_0 g_1 \bigg [ -\alpha I +\left( 2 \alpha (m_1^2-m_2^2) - 2 \beta m_0 m_2 \right) I_0\nonumber \\&+\, \left( \alpha (-m_0^2-m_1^2-m_2^2) - 2 \beta m_0 m_2 \right) I_1\nonumber \\&+\, \bigg ( 2 \alpha \left( (m_1^2-m_2^2)^2-m_0^4 \right) \nonumber \\&-\, 4 \beta m_0 m_2 (m_0^2+m_1^2-m_2^2)\bigg ) I_{10} \bigg ]\nonumber \\&+\, g_1^2 \bigg [ \alpha I +\left( \alpha (-m_0^2+3 m_1^2-m_2^2) - 2 \beta m_0 m_2 \right) I_1\nonumber \\&+\, \bigg ( -2\alpha (m_0^2-m_1^2+m_2^2) m_1^2 \nonumber \\&-\, 4 \beta m_0 m_2 m_1^2\bigg ) I_{11}\bigg ]\nonumber \\&+\, g_0 g_2 \bigg [ -2 \alpha I +\left( 2 \alpha (m_1^2-m_2^2) - 2 \beta m_0 m_2 \right) I_0\nonumber \\&+\, \left( -2\alpha (m_0^2-m_2^2) - 2 \beta m_0 m_2 \right) I_2\nonumber \\&+\, \bigg (- 2 \alpha (m_0^2-m_1^2+m_2^2)^2 \nonumber \\&-\, 4 \beta m_0 m_2 (m_0^2-m_1^2+m_2^2)\bigg ) I_{20} \bigg ]\nonumber \\&+\, g_1 g_2 \bigg [ \alpha I +\left( \alpha (m_0^2+m_1^2+m_2^2)+2 \beta m_0 m_2 \right) I_1\nonumber \\&+\, \left( 2\alpha (m_0^2-m_1^2)+ 2 \beta m_0 m_2 \right) I_2\nonumber \\&+\, \bigg ( \alpha \left( 2 m_2^4-2(m_0^2-m_1^2)^2 \right) \nonumber \\&+\, 4 \beta m_0 m_2 (-m_0^2+m_1^2+m_2^2)\bigg ) I_{21} \bigg ]\nonumber \\&+\, g_2^2 \bigg [ \alpha I + (-2 \alpha m_2^2 - 2 \beta m_0 m_2 )I_2 +\alpha I_2^1\nonumber \\&+\, \left( -2\alpha (m_0^2-m_1^2+m_2^2) m_2^2 -4 \beta m_0 m_2^3\right) I_{22}\bigg ],\nonumber \\ \end{aligned}$$

where $\alpha = 2 g_s^2 (|U^{\tilde{q}}_{i,g}|^2+|U^{\tilde{q}}_{i,g+3}|^2)$ and $\beta = - 4 g_s^2 \mathrm{Re}\big (U^{\tilde{q}*}_{i,g}\, U^{\tilde{q}}_{i,g+3} \big )$. Note that the spin average for the incoming fermion of 1/2 is already included. For the gluino decays *col* is 1/8.

The explicit result for photon radiation is

25 $$\begin{aligned} \Gamma (\tilde{g}\rightarrow \tilde{u}_i u_g \gamma ) = {1 \over 512 \pi ^3 m_{\tilde{g}}}\, 4 \,X_\mathrm{FSF}, \end{aligned}$$

taking in $X_\mathrm{FSF}$, Eq. (24), $g_0 = 0, g_1 = -e Q_1, g_2 = -e Q_2$, *e* denotes the positron charge and $Q_{1,2}$ the charge of the particle on leg 1 or 2 in units of *e*, respectively. The additional factor 4 stems from the colour summation, which is universal, $\mathrm{Tr}(T^a T^a) = 3 C_F = 4$.

The result for gluon radiation reads

26 $$\begin{aligned} \Gamma (\tilde{g}\rightarrow \tilde{u}_i u_g g) = {1 \over 512 \pi ^3 m_{\tilde{g}}} X_\mathrm{FSF}. \end{aligned}$$

In this case the colour summation results in the $3 \times 3$ matrix *C*. We take in $X_\mathrm{FSF}$, Eq. (24), $g_i g_j \rightarrow g_{si}g_{sj}C_{ij}$, where $g_{si}= g_s Q_{si}$, $g_s =\sqrt{4 \pi \alpha _s}$ is the strong coupling constant and $Q_{si}=\pm 1$ is the colour charge factor for particles carrying colour/anti-colour, respectively. The matrix *C* describes the colour traces of the SU(3)$_C$ generators and is given by

27 $$\begin{aligned} C = \left( \begin{array}{l@{\quad }l@{\quad }l} 12 &{} 6 &{} -6 \\ 6 &{} 16/3 &{} -2/3 \\ -6 &{} -2/3 &{} 16/3 \\ \end{array} \right) , \end{aligned}$$

e.g. $$C_{00} = 12$$.

## Footnotes

1 Note, that in the $\overline{\mathrm{DR}}$ scheme the coupling corrections contain only UV divergent terms which have to be cancelled exactly to yield a convergent result.

2 A comparison with the tree-level results is not precisely accurate, since in the $\overline{\mathrm{DR}}$ scheme the tree-level width alone does not have a physical meaning, but the width at full one-loop level does. However, in order to get approximately an idea how large the tree-level result is, we allow to use on-shell masses only in the kinematics factor of Eq. (12). In the following, we will call this tree-level result.
